# Squamous cell carcinoma of the rectum: Practice trends and patient survival

**DOI:** 10.1002/cam4.1893

**Published:** 2018-11-20

**Authors:** Sunil W. Dutta, Clayton E. Alonso, Mark R. Waddle, Shiv R. Khandelwal, Einsley‐Marie Janowski, Daniel M. Trifiletti

**Affiliations:** ^1^ Department of Radiation Oncology University of Virginia Charlottesville Virginia; ^2^ Department of Radiation Oncology Mayo Clinic Jacksonville Florida

**Keywords:** cancer, chemoradiation, National Cancer Database, radiation, rectal, squamous

## Abstract

**Purpose:**

Leverage the National Cancer Database (NCDB) to evaluate trends in management of nonmetastatic squamous cell cancer (SCC) of the rectum and their effect on survival for this uncommon tumor.

**Methods and Materials:**

Retrospective data was obtained from the NCDB for patients diagnosed with SCC of the rectum between 2004 and 2014, including cT1‐4, cN0‐2, cM0 tumors (cohort A, n = 2296). A subgroup analysis was performed on locally advanced tumors (cT1‐T2, N+ or cT3, N any, subcohort B, n = 883), treated with chemoradiation (n = 706) or trimodality therapy (n = 177) including chemotherapy, radiation, and surgery. Pathological complete response rate following neoadjuvant therapy was obtained. Univariate and multivariate logistic regression analyses were performed to generate hazard ratios (HR) investigating factors associated with overall survival. Kaplan‐Meier (K‐M) method was used to estimate overall surviving proportion at 5 and 10 years.

**Results:**

The K‐M estimated 5 and 10 year overall survival for stage I disease was 71.3% and 57.8%, respectively; stage II disease was 57.0% and 38.9%, respectively; stage III disease was 57.8% and 41.5%, respectively. On multivariate analysis, higher cT category (*P* < 0.001) resulted in worse survival. For locally advanced tumors (subcohort B), there was no significant difference in survival between chemoradiation alone compared to trimodality therapy (*P* = 0.909 on multivariate analysis).

**Conclusions:**

Most providers manage locally advanced SCC of the rectum similar to anal cancer, which results in equivalent overall survival and spares patients from the additional morbidity associated with surgical resection.

## 1 INTRODUCTION

Squamous cell carcinoma (SCC) of the gastrointestinal (GI) tract most commonly occurs in the esophagus or anal canal, and prior studies report a <1% incidence within the rectum.^1^ Due to its rarity, the etiology of SCC of the rectum remains unclear, although it has been linked to chronic inflammation and prior radiotherapy.^2^, ^3^, ^4^, ^5^ A recent Surveillance, Epidemiology, and End Results (SEER) analysis showed those with SCC to have a favorable prognosis compared to adenocarcinoma of the rectum.^6^ While small, noninvasive tumors within the rectum can be managed with conservative measures such as surgery alone, more advanced rectal tumors often benefit from further intervention, including chemotherapy and/or radiation.^7^ Currently, no consensus guidelines exist for the treatment of nonmetastatic rectal cancer with SCC histology, which may be misguided considering its optimal treatment may differ from adenocarcinoma of the rectum. For example, the National Comprehensive Cancer Network recognizes mucosal melanoma of the GI tract as a separate entity entirely.^8^

Additionally, treatment of SCC of the anal canal has been shown to be managed markedly differently from rectal adenocarcinoma, with combined intensive chemotherapy and radiation without planned surgery being standard of care for locoregional anal SCC tumors, as reported by Nigro et al.^9^ The purpose of this study was to leverage the National Cancer Database (NCDB) to evaluate current trends in management and their effect on survival for this uncommon tumor. While the NCDB lacks local recurrence rates, unsalvageable recurrences result in reduced survival. The large patient numbers available with NCDB analysis should allow us to determine whether any survival detriment based on treatment allocation exists.^10^

## 2 METHODS AND MATERIALS

### 2.1 Data source

The NCBD, established in 1989, is a nationally recognized clinical oncology database sponsored by the American College of Surgeons and the American Cancer Society. The NCDB collects data from more than 1500 facilities accredited by the Commission on Cancer and contains information on treatments and outcomes for patients with malignant disease. The current database gathers more than 70% of new cancer diagnoses in the United States and contains more than 34 million historical records.^11^

**Figure 1:**
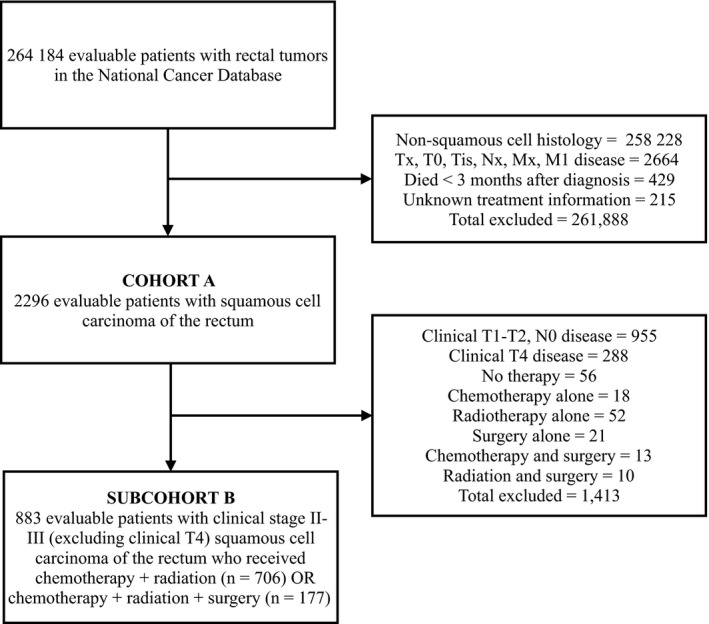
Cohort selection diagram

Data were obtained from the NCDB for patients diagnosed with rectal cancer between 2004 and 2014 (264, 184 patients). We limited patients to squamous cell histology (histology codes 8070‐8083, 258 636 patients excluded). Patients with incomplete staging information or metastatic disease were excluded (2664 excluded). We excluded patients who died within 3 months of diagnosis due to competing risks of noncancer‐related deaths (eg myocardial infarction; 429 excluded). Patient with unknown receipt of chemotherapy, radiation, or surgery were also excluded (215 excluded). While specific surgical technique is unavailable, surgery, if performed, was defined by the NCDB as definitive. Figure 1 shows the complete selection diagram with 261 888 total patients excluded.

The remaining 2296 patients, defined as cohort A, included cT1‐4, cN0‐2, cM0 SCC rectal tumors, according to American Joint Committee on Cancer (AJCC); these patients were then analyzed based on available database information.^12^ An additional subgroup, called subcohort B, was further filtered to only include cT1‐T3, cN+ or cT3, cN any SCC rectal tumors, which represent locally advanced tumors that, under current rectal cancer guidelines, include trimodality therapy as standard of care (chemotherapy, radiation, and surgery).^8^ To compare modalities, the two most common treatment approaches were included in the final analysis of subcohort B: chemotherapy and radiation or trimodality (chemotherapy, radiation therapy, and surgical resection). Patients with clinical T4 tumors were excluded from cohort B as those rectal tumors are less amenable to resection and can vary in their treatment sequence (eg neoadjuvant chemotherapy followed by restaging, then definitive local therapy).^8^, ^13^

### 2.2 Statistical analyses

The primary outcome measure of this study for each cohort was the overall survival of patients with nonmetastatic squamous cell carcinoma of the rectum. Important prognostic factors that may influence treatment or outcome, including gender, age, race, median income of zip code, distance to hospital, facility type, Charlson/Deyo score, tumor category, nodal category, receipt of chemotherapy, receipt of radiation, and receipt of surgery, were evaluated. A secondary outcome measure was pathological complete response rate among patients in each cohort.

Univariate and multivariate analyses (log rank, Cox regression, and binary logistic models) were performed to generate hazard ratios (HR) to investigate factors associated with overall survival. Potential prognostic variables in the multivariate models were chosen through purposeful selection and univariate analyses to investigate significance. Factors associated with a *P* < 0.10 on univariate analysis were included in the multivariate models. Kaplan‐Meier was used to estimate survival at 5 and 10 years. All statistical analyses were performed using the SPSS program (SPSS, version 24.0; SPSS Inc, Chicago, IL), and *P* < 0.05 on multivariate analysis was considered statistically significant.

## 3 RESULTS

### 3.1 Clinical and treatment characteristics of cohort A (cT1‐T4, cN0‐N2, cM0 SCC of the rectum)

After planned exclusions, 2296 patients with nonmetastatic SCC of the rectum were identified from the NCDB database (Figure 1). Table 1 describes the clinical characteristics of cohort A. There was a strong female predilection (70.8% female). Most patients identified themselves as Caucasian (81.6%), followed by African American (10.3%) and Hispanic (5.7%). The majority of patients had few comorbidities (Charlson/Deyo score = 0, 81.2%). Most patients were managed through a comprehensive community cancer program (43.3%). The remaining patients underwent therapy at academic or research facilities (30.8%), integrated network cancer programs (12.2%), and community cancer programs (11.1%). The majority of patients had cT3 (35.8%) and cN0 (66.9%) disease. Regarding AJCC 7th edition staging for rectal cancer, 955 (41.6%) were stage I, 582 (25.3%) were stage II, and 759 (33.1%) were stage III. There was an increase in recorded diagnoses over time, from 149 cases to in 2004 to 307 cases in 2014. The majority of patients received chemotherapy (79.8%) and/or radiation (82.1%), and only 27.3% of patients underwent surgery.

**Table 1:** Clinical and treatment characteristics of 2296 patients (cohort A) with nonmetastatic squamous cell cancer of the rectum in the National Cancer Database (2004‐2014)

	n or median	% or range
Clinical characteristics		
Age (years old, median)	60	20‐90
Sex		
Male	670	29.2
Female	1626	70.8
Race		
White	1874	81.6
African American	237	10.3
American Indian	4	0.2
Asian/Pacific Islander	21	0.9
Unknown	30	1.3
Hispanic	130	5.7
Charlson/Deyo score		
0	1865	81.2
1	294	12.8
2	60	2.6
3	77	3.4
Median income of zip		
<$38 000	464	20.2
$38 000‐$47 999	532	23.2
$48 000‐$62 999	619	27.0
≥$63 000	659	28.7
Distance to Hospital		
<25 miles	1880	81.9
25‐100 miles	326	14.2
>100 miles	68	3.0
Facility type		
CCP	254	11.1
CCCP	995	43.3
Academic/research	708	30.8
INCP	281	12.2
Year of diagnosis		
2004	149	6.5
2005	120	5.2
2006	137	6.0
2007	183	8.0
2008	190	8.3
2009	218	9.5
2010	216	9.4
2011	254	11.1
2012	266	11.6
2013	256	11.1
2014	307	13.4
Disease characteristics		
cTcategory		
cT1	592	25.8
cT2	595	25.9
cT3	821	35.8
cT4	288	12.5
cN category		
cN0	1537	66.9
cN1	567	24.7
cN2	192	8.4
Clinical stage		
I	955	41.6
II	582	25.3
III	759	33.1
Tumor grade		
Well differentiated	121	5.3
Moderately differentiated	757	33.0
Poorly differentiated	767	33.4
Undifferentiated	26	1.1
Unknown	625	27.2
Treatment characteristics		
Chemotherapy		
No	464	20.2
Yes	1832	79.8
Radiotherapy		
No	411	17.9
Yes	1885	82.1
Surgery		
No	1670	72.7
Yes	626	27.3

CCCP, comprehensive community cancer program; CCP, community cancer center; CI, confidence interval; HR, hazard ratio; INCP, integrated network cancer program.

### 3.2 Overall survival analysis of cohort A (cT1‐T4, cN0‐N2, cM0 SCC of the rectum)

Univariate and multivariate analyses were performed to investigate factors associated with overall survival, for which the complete results are shown in Table 2. On univariate analysis of patient characteristics, patients with increasing age, male gender, African American race, higher Charlson/Deyo score, and lower median income of zip code were all associated with worse overall survival (*P* < 0.05). On multivariate analysis of patient characteristics, increasing age (*P* < 0.001), male gender (HR = 0.639 for females, compared to male, *P* < 0.001), and higher Charlson/Score (*P* < 0.05) remained significant.

**Table 2:** Analysis of factors associated with overall survival following diagnosis for 2296 patients (cohort A) with nonmetastatic squamous cell cancer of the rectum in the National Cancer Database (2004‐2014)

	Univariate	Multivariate		
	*P*‐value	*P*‐value	HR	95% CI
Clinical characteristics				
Age (years old)	**<0.001**	**<0.001**	**1.031**	**1.025‐1.037**
Sex	**<0.001**			
Male			ref	
Female		**<0.001**	**0.639**	**0.549‐0.745**
Race	**0.020**			
White			ref	
Black		0.344	1.120	0.886‐1.416
American Indian		0.663	0.645	0.090‐4.626
Asian/Pacific Islander		0.941	1.027	0.508‐2.079
Unknown		0.136	1.529	0.874‐2.674
Hispanic		0.376	1.148	0.846‐1.558
Charlson/Deyo score	**<0.001**	**<0.001**		
0			ref	
1		**0.002**	**1.367**	**1.117‐1.672**
2		**<0.001**	**2.146**	**1.522‐3.028**
3		**<0.001**	**2.197**	**1.548‐3.117**
Median income of zip	**<0.001**	**<0.001**		
<$38 000			ref	
$38 000‐$47 999		**0.038**	**0.804**	**0.654‐0.988**
$48 000‐$62 999		0.114	0.850	0.696‐1.040
≥$63 000		**<0.001**	**0.612**	**0.495‐0.757**
Distance to Hospital	0.261			
<25 miles				
25‐100 miles				
>100 miles				
Facility type	0.927			
CCP				
CCCP				
Academic/Research				
INCP				
Disease characteristics				
cTcategory	**<0.001**	**<0.001**		
cT1			ref	
cT2		0.117	0.829	0.655‐1.048
cT3		**<0.001**	**1.551**	**1.258‐1.912**
cT4		**<0.001**	**2.561**	**2.019‐3.249**
cN category	**0.049**	0.088		
cN0			ref	
cN1		0.063	1.184	0.991‐1.415
cN2		0.060	1.290	0.989‐1.682
Tumor grade	0.240			
Well differentiated				
Moderately differentiated				
Poorly differentiated				
Undifferentiated				
Unknown				
Treatment characteristics				
Chemotherapy	**<0.001**			
No			ref	
Yes		**<0.001**	**0.531**	**0.417‐0.676**
Radiotherapy	**<0.001**			
No			ref	
Yes		0.095	0.804	0.622‐1.039
Surgery	**0.026**			
No			ref	
Yes		**0.002**	**0.758**	**0.639‐0.900**

CCCP, comprehensive community cancer program; CCP, community cancer center; CI, confidence interval; HR, hazard ratio; INCP, integrated network cancer program; RT, radiation therapy.

Numbers bolded for *P* < 0.05.

**Figure 2:**
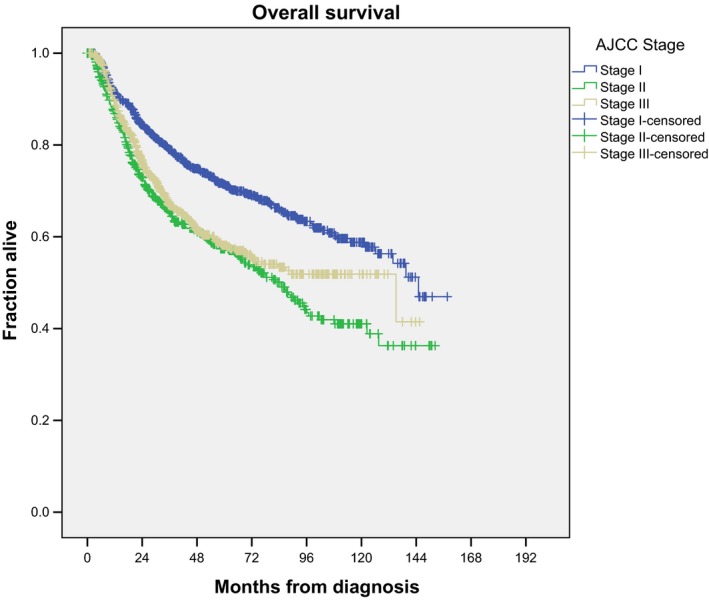
Unadjusted Kaplan‐Meier curve for each stage of disease within cohort A, per American Joint Committee on Cancer 7th edition staging criteria

Regarding disease characteristics, higher cT category (*P* < 0.001) and cN category (*P* = 0.049) were associated with worse survival on univariate analysis. Notably, tumor grade did not influence survival. On multivariate analysis, only higher cT category remained significant (HR = 1.551 and 2.561 for cT3 and cT4 tumors compared to cT1, respectively, *P* < 0.001 for each). Figure 2 shows the unadjusted Kaplan‐Meier curve for each stage of disease, per AJCC 7th edition staging criteria. The Kaplan‐Meier estimated 5‐ and 10‐year survival for stage I disease was 71.3% and 57.8%. The Kaplan‐Meier estimated 5‐ and 10‐year survival for stage II disease was 57.0% and 38.9%. The Kaplan‐Meier estimated 5‐ and 10‐year survival for stage III disease was 57.8% and 41.5%, respectively. Notably, while there was a difference in survival from stage I to stage II/III disease (log rank *P* < 0.001 for each), no survival difference was seen between stage II and stage III disease (log rank *P* = 0.119), likely due to no difference in survival among nodal category with SCC histology.

Regarding treatment characteristics, all treatment modalities (chemotherapy, radiotherapy, and surgery) were associated with improved survival on univariate analysis. On multivariate, only receipt of chemotherapy (HR = 0.531, *P* < 0.001) and surgery (HR = 0.758, *P* = 0.002) were associated with improved overall survival for cohort A.

### 3.3 Overall survival analysis of subcohort B (cT1‐T2, cN+, cM0 or cT3, cN any, cM0 SCC of the rectum)

To better clarify the optimal management of locally advanced tumors where the current standard of care for rectal cancer includes trimodality therapy, patients were filtered to only include clinical stage II and III disease (excluding cT4) which resulted in 1053 patients. Among these patients, the majority either received chemotherapy and radiation (n = 706, 67%) or chemotherapy, radiation, and surgery (n = 177, 16.8%). Less common combined modalities utilized were chemotherapy and surgery (n = 13, 1.2%) and radiation and surgery (n = 10, 0.9%). To compare the two most common modalities, other regimens were excluded from the final subcohort B survival analysis (Figure 1), resulting in 883 patients with complete evaluable treatment information (Table 3).

**Table 3:** Analysis of factors associated with overall survival following diagnosis for 883 patients (subcohort B) with locally advanced (cT1‐T2, cN+, cM0 or cT3, cN any, cM0) squamous cell cancer of the rectum in the National Cancer Database (2004‐2014)

	Univariate	Multivariate		
	*P*‐value	*P*‐value	HR	95% CI
Clinical characteristics				
Age (years old)	**0.002**	**0.001**	**1.019**	**1.008‐1.030**
Sex	**0.002**			
Male			ref	
Female		**0.015**	**0.718**	**0.550‐0.937**
Race	0.684			
White				
Black				
American Indian				
Asian/Pacific Islander				
Unknown				
Hispanic				
Charlson/Deyo score	**<0.001**	**<0.001**		
0			ref	
1		0.792	.949	0.640‐1.405
2		**0.022**	**1.901**	**1.096‐3.296**
3		**0.034**	**1.785**	**1.044‐3.051**
Median income of zip	**0.001**	**0.008**		
<$38 000			ref	
$38 000‐$47 999		0.094	0.747	0.532‐1.051
$48 000‐$62 999		0.128	0.777	0.561‐1.075
≥$63 000		**0.001**	**0.544**	**0.384‐0.770**
Distance to Hospital	0.588			
<25 miles				
25‐100 miles				
>100 miles				
Facility type	0.682			
CCP				
CCCP				
Academic/Research				
INCP				
Disease characteristics				
cTcategory	**0.001**			
cT1			ref	
cT2		0.723	0.877	0.424‐1.814
cT3		0.174	1.556	0.822‐2.945
cN category	0.375			
cN0				
cN1				
cN2				
Tumor grade	0.353			
Well differentiated				
Moderately differentiated				
Poorly differentiated				
Undifferentiated				
Unknown				
Treatment characteristics				
Therapy type	0.936			
Chemo + RT				
Chemo + RT + Surgery		0.909	0.983	0.734‐1.317

CCCP, comprehensive community cancer program; CCP, community cancer center; CI, confidence interval; HR, hazard ratio; INCP, integrated network cancer program; RT, radiation therapy.

Numbers bolded for *P* < 0.05.

**Figure 3:**
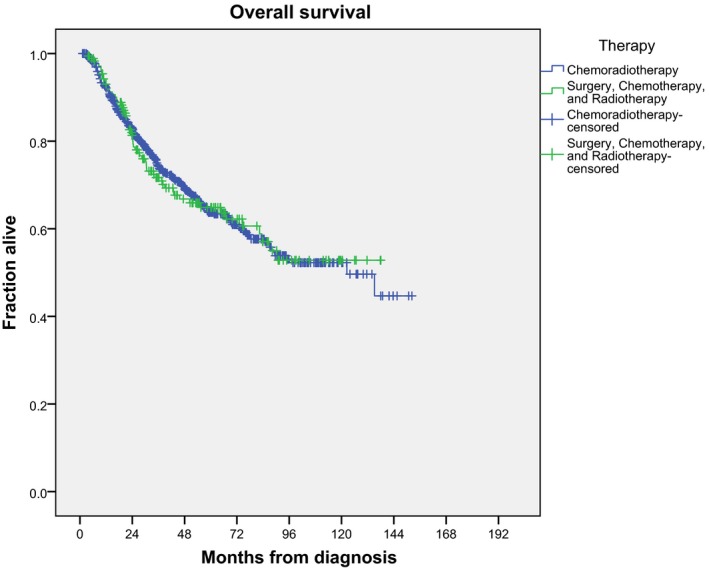
Unadjusted Kaplan‐Meier curve of overall survival from time of diagnoses for patients receiving either chemotherapy and radiation or trimodality therapy (chemotherapy, radiation, and chemotherapy) for locally advanced squamous cell cancer of the rectum (subcohort B). No survival difference was observed (*P = *0.304 by log rank)

On univariate analysis of subcohort B, younger age, female gender, lower Charlson‐Deyo score, lower cT category, and trimodality therapy (versus chemotherapy and radiation) were associated with improved overall survival (*P* < 0.05 for each). On multivariate analysis, older age (HR = 1.019 per year, *P* = 0.001), higher Charlson/Deyo scores (*P* < 0.05), and lower median income of zip (*P* = 0.001) were associated with worse survival. Of note, therapy type, comparing chemotherapy, and radiation to trimodality therapy (chemotherapy, radiation, and surgery) showed no significant difference in survival (*P* = 0.909 on multivariate analysis). Figure 3 shows the unadjusted Kaplan‐Meier curve for subcohort B, separated by therapy type. The Kaplan‐Meier estimated 5‐ and 10‐year survival for entire cohort B is 63.8% and 50.3% (log rank *P* = 0.936 between receipt of chemotherapy and radiation ± surgery).

### 3.4 Pathological response to therapy

Among cohort A, who underwent radiation therapy prior to surgery and have available pathology information, 36.0% (41/114 patients) experienced a complete pathological response. 39/41 (95.1%) of those patients with a complete pathological response also received neoadjuvant chemotherapy prior to surgery, in addition to radiation. The median number of days from end of radiation to definitive surgery for those with a complete pathological response (p0) was 67 days (n = 38, range 9‐237 days) versus 73 days (n = 70, range 6‐320 days) for those without a complete response (p1‐4 disease) on surgical pathology (*P* = 0.29). The median regional radiation dose was 45 Gy with a boost administered to 46.3% of patients (median 5.4 Gy). While univariate analysis demonstrated a trend toward a survival advantage with complete pathological responders (*P = *0.085), it was not significant on multivariate analysis (*P = *0.110).

Among patients in subcohort B who underwent radiation therapy prior to surgery and have available pathology information, 41.1% (30/73 patients) experienced a complete pathological response on surgical pathology. No survival advantage was observed comparing pathologic complete versus noncomplete responders in subcohort B (*P = *0.129 on univariate analysis).

## 4 DISCUSSION

This database analysis reveals notable differences between SCC and adenocarcinoma of the rectum. Patients with rectal SCC present more often as slightly younger, female patients, who may benefit from the avoidance of surgery to reduce additional long term treatment related morbidity.^14^ For example, patients with clinical stage II or III disease (excluding cT4) experienced no difference in survival between chemoradiation and trimodality therapy (chemotherapy, radiation, and surgery). Additionally, for those treated with neoadjuvant therapy, the pathological complete response was 36%‐41%. The timing of the operations indicates that these are planned surgeries, but hints at potential treatment response to chemoradiation. Due to the rarity of this tumor, prospective trials to compare these modalities are unlikely to occur, and, therefore, this database analysis sheds light on the unique outcome of SCC of the rectum.

The SEER study performed by Chiu et al^6^ helped establish epidemiology and outcomes of rectal SCC, including a female predominance and a more favorable prognosis compared to rectal adenocarcinoma. Similar to our study, authors showed that receipt of surgery did not impact overall survival. Their study is limited in that they were unable to address chemotherapy utilization and pathological response rates. Furthermore, while they grouped patients into risk groups, specific TNM information was not available or reported, limiting its application to managing certain stages of disease. It should be noted that, unlike rectal adenocarcinoma, nodal category has less impact on survival compared to tumor category, consistent with a prior review.^15^ Reasons we hypothesize for this finding include difficulty in accurately assessing nodal status clinically, unique history of SCC of the rectum nodal metastases, or treatment strategies currently in use are effective in controlling nodal disease with SCC histology.

We specifically created subcohort B that, under rectal cancer guidelines, may be managed with trimodality therapy, which is the current standard of care. However, our study supports the effectiveness of chemoradiation, without surgery. The NCDB (and SEER analyses) are unable to detect rates of local recurrence and salvage surgery for those who do not receive surgery as part of their initial treatment course. Regardless, even if a substantial portion of patients ultimately require salvage surgery, our data do not show a survival benefit to adding surgery during the initial course. This is consistent with a prior report by Kulaylat et al who grouped stage I‐III patients and found that those with SCC of the anus requiring salvage surgery (ie ≥12 weeks after chemoradiation) had worse survival, compared to no survival difference for those with SCC of the rectum receiving salvage surgery. Taken in conjunction with the present study, planned or late surgery for SCC of the rectum results in no survival benefit after chemoradiation.^16^

While the NCDB does not provide specific chemotherapy agents, there is a growing body of literature from individual institutions showing good outcomes with agents used for the treatment of anal SCC.^17^ Clark et al reported seven cases treated using the Anal Cancer Trial II (ACT II) protocol, which included 5.5 weeks of radiation with concurrent 5‐flurouracil and mitomycin C or cisplatin.^18^, ^19^ At a median follow‐up of 18 months, no recurrences were noted, with only one patient undergoing surgery, which showed no viable tumor on surgical pathology. Sturgeon et al^20^ reported outcomes for 14 patients with SCC of the rectum who underwent similar definitive chemoradiation using an anal cancer regimen. With a median follow‐up of 4.5 years, only 3/14 patients recurred, and two were successfully managed with salvage surgery. Musio et al^21^ reported outcomes for eight patients treated with definitive management similar to anal cancer; their study reported a negative biopsy in 75% of patients 6 months after chemoradiation, and only one patient required a salvage surgery at the end of treatment. For those that received surgery, our study reports a short interval between radiation and surgery (median 69 days for cohort A), indicating that the surgery was planned and not performed as salvage. Additional time from completion of radiation may have resulted in higher pathological complete response rates, with data from the ACT II trial suggesting that 182 days is the optimal time to assess response in anal SCC.^22^ Unfortunately, surveillance for rectal SCC would involve endoscopy to evaluate tumor response, whereas anal cancer can be more easily examined with inspection or digital rectal exam. Also, proximal versus distal tumor location is not provided by the NCDB, limiting further analysis. However, it should be noted that the normal tissue (ie skin) around the anus is more sensitive to radiation, and it may be possible to treat less distal tumors with higher doses of radiation to gross disease, further improving the tumor response rate.^23^ Most patients in our series received a moderate dose of 45‐50.4 Gy.

In summary, existing literature shows that chemotherapy regimens utilized for anal cancer (eg 5‐flurouracil and mitomycin C) given concurrently with radiation provides a durable response and that patients do well with salvage surgery, if needed. This is exemplified with our data, showing similar survival for patients with stage II‐III (excluding T4) cancer managed with chemoradiation. While our NCDB analysis does not provide chemotherapy agents used, the current literature supports regimens used in anal cancer.^24^ One hypothesis for the lower pathological complete response rate in our study compared to institutional reports is that more effective, albeit more toxic, regimens used in anal cancer (eg mitomycin C) given concurrently with radiation may have been substituted with a more tolerable concurrent chemotherapy agent (eg capecitabine), with the expectation that the patient will undergo definitive resection.

Limitations to our study should be noted. As discussed, the NCDB is limited by nonrandom allocation to treatment modality and the lack of detailed information regarding medical comorbidities, both of which may obscure comparisons of treatment methods through the introduction of confounding. We performed multivariate analysis to adjust for measured potential confounders, but significant potential biases remain. Furthermore, chemotherapy agents and extent (ie number of cycles) is not known. However, we excluded patients with incomplete staging and unknown treatment information, therefore strengthening the reliability of the outcome for each treatment group. Lastly, disease‐specific outcomes including local recurrence and salvage surgery rate is unknown.

## 5 CONCLUSION

This database analysis shows that most providers are managing locally advanced rectal SCC similar to anal cancer, which results in equivalent overall survival and spares patients from the additional morbidity associated with surgical resection. While most surgeries were presumably planned, a pathological complete response rate up to 41% suggests radiation and chemotherapy has an effective response.

## CONFLICT OF INTEREST

None.

## References

[1] Kang H , O'Connell JB , Leonardi MJ , et al. Rare tumors of the colon and rectum: a national review. Int J Colorectal Dis. 2007;22(2):183‐189. 16845516 10.1007/s00384-006-0145-2

[2] Scaringi S , Bisogni D , Messerini L , et al. Squamous cell carcinoma of the middle rectum: report of a case and literature overview. Int J Surg Case Rep. 2015;7C:127‐129. 25465645 10.1016/j.ijscr.2014.10.097 PMC4336389

[3] Leung KK , Heitzman J , Madan A . Squamous cell carcinoma of the rectum 21 years after radiotherapy for cervical carcinoma. Saudi J Gastroenterol. 2009;15(3):196‐198. 19636183 10.4103/1319-3767.54745 PMC2841421

[4] Cheng H , Sitrin MD , Satchidanand SK , et al. Colonic squamous cell carcinoma in ulcerative colitis: report of a case and review of the literature. Can J Gastroenterol. 2007;21(1):47‐50. 17225882 10.1155/2007/904081 PMC2656630

[5] Zirkin RM , McCord DL . Squamous cell carcinoma of the rectum: report of a case complicating chronic ulcerative colitis. Dis Colon Rectum. 1963;6:370‐373. 14063163 10.1007/BF02618400

[6] Chiu MS , Verma V , Bennion NR , et al. Comparison of outcomes between rectal squamous cell carcinoma and adenocarcinoma. Cancer Med. 2016;5(12):3394‐3402. 27781400 10.1002/cam4.927 PMC5224838

[7] Glimelius B , Tiret E , Cervantes A , et al. Rectal cancer: ESMO clinical practice guidelines for diagnosis, treatment and follow‐up. Ann Oncol. 2013;24(suppl 6):vi81‐vi88. 24078665 10.1093/annonc/mdt240

[8] National Comprehensive Cancer Network .Rectal cancer (Version 1.2018). https://www.nccn.org/professionals/physician_gls/pdf/rectal.pdf. Accessed March 13, 2018; April 2, 2018.

[9] Nigro ND , Seydel HG , Considine B , et al. Combined preoperative radiation and chemotherapy for squamous cell carcinoma of the anal canal. Cancer. 1983;51(10):1826‐1829. 6831348 10.1002/1097-0142(19830515)51:10<1826::aid-cncr2820511012>3.0.co;2-l

[10] Cai Y , Li Z , Gu X , et al. Prognostic factors associated with locally recurrent rectal cancer following primary surgery (Review). Oncol Lett. 2014;7(1):10‐16. 24348812 10.3892/ol.2013.1640 PMC3861572

[11] American College of Surgeons, Q.P .National cancer database. https://www.facs.org/quality-programs/cancer/ncdb. Accessed December 7, 2017.

[12] American College of Surgeons . National cancer database: patient user file data dictionary items. http://ncdbpuf.facs.org/?q=node/259. Accessed December 7, 2017.

[13] Wu JS . Rectal cancer staging. Clin Colon Rectal Surg. 2007;20(3):148‐157. 20011196 10.1055/s-2007-984859 PMC2789513

[14] Bokey EL , Chapuis PH , Hughes WJ , et al. Morbidity, mortality and survival following resection for carcinoma of the rectum at Concord Hospital. Aust N Z J Surg. 1990;60(4):253‐259. 2322212 10.1111/j.1445-2197.1990.tb07363.x

[15] Kassir R , Baccot S , Bouarioua N , et al. Squamous cell carcinoma of middle rectum: literature review. Int J Surg Case Rep. 2014;5(2):86‐90. 24441443 10.1016/j.ijscr.2013.12.011 PMC3921652

[16] Kulaylat AS , Hollenbeak CS , Stewart DB Sr . Squamous cancers of the rectum demonstrate poorer survival and increased need for salvage surgery compared with squamous cancers of the anus. Dis Colon Rectum. 2017;60(9):922‐927. 28796730 10.1097/DCR.0000000000000881

[17] Ghosn M , Kourie HR , Abdayem P , et al. Anal cancer treatment: current status and future perspectives. World J Gastroenterol. 2015;21(8):2294‐2302. 25741135 10.3748/wjg.v21.i8.2294 PMC4342904

[18] James RD , Glynne‐Jones R , Meadows HM , et al. Mitomycin or cisplatin chemoradiation with or without maintenance chemotherapy for treatment of squamous‐cell carcinoma of the anus (ACT II): a randomised, phase 3, open‐label, 2 x 2 factorial trial. Lancet Oncol. 2013;14(6):516‐524. 23578724 10.1016/S1470-2045(13)70086-X

[19] Clark J , Cleator S , Goldin R , et al. Treatment of primary rectal squamous cell carcinoma by primary chemoradiotherapy: should surgery still be considered a standard of care? Eur J Cancer. 2008;44(16):2340‐2343. 18707873 10.1016/j.ejca.2008.07.004

[20] Sturgeon JD , Crane CH , Krishnan S , et al. Definitive chemoradiation for squamous cell carcinoma of the rectum. Am J Clin Oncol. 2017;40(2):163‐166. 25222072 10.1097/COC.0000000000000126

[21] Musio D , De Felice F , Manfrida S , et al. Squamous cell carcinoma of the rectum: the treatment paradigm. Eur J Surg Oncol. 2015;41(8):1054‐1058. 25956212 10.1016/j.ejso.2015.03.239

[22] Glynne‐Jones R , Sebag‐Montefiore D , Meadows HM , et al. Best time to assess complete clinical response after chemoradiotherapy in squamous cell carcinoma of the anus (ACT II): a post‐hoc analysis of randomised controlled phase 3 trial. Lancet Oncol. 2017;18(3):347‐356. 28209296 10.1016/S1470-2045(17)30071-2 PMC5337624

[23] Gunther JR , Chadha AS , Shin US , et al. Preoperative radiation dose escalation for rectal cancer using a concomitant boost strategy improves tumor downstaging without increasing toxicity: a matched‐pair analysis. Adv Radiat Oncol. 2017;2(3):455‐464. 29114614 10.1016/j.adro.2017.04.001 PMC5605486

[24] Glynne‐Jones R , Nilsson PJ , Aschele C , et al. Anal cancer: ESMO‐ESSO‐ESTRO clinical practice guidelines for diagnosis, treatment and follow‐up. Radiother Oncol. 2014;111(3):330‐339. 24947004 10.1016/j.radonc.2014.04.013

